# Interleukin-24-mediated antitumor effects against human glioblastoma via upregulation of P38 MAPK and endogenous TRAIL-induced apoptosis and LC3-II activation-dependent autophagy

**DOI:** 10.1186/s12885-023-11021-y

**Published:** 2023-06-06

**Authors:** Seyedeh Maliheh Babazadeh, Mohammad Reza Zolfaghari, Mohsen Zargar, Kazem Baesi, Sayed Younes Hosseini, Amir Ghaemi

**Affiliations:** 1 Department of Microbiology, Faculty of Basic Science, Qom Branch, Islamic Azad University, Qom, Iran; 2 Hepatitis and AIDS Department, Pasteur Institute of Iran, Tehran, Iran; 3 Bacteriology and Virology Department, School of Medicine, Shiraz University of Medical Sciences, Shiraz, Iran; 4 Department of Influenza and other Respiratory Viruses, Pasteur Institute of Iran, Tehran, Iran

**Keywords:** IL-24, Recombinant adenovirus, Cell apoptosis, Glioblastoma, Antitumor, Autophagy

## Abstract

**Background:**

Melanoma differentiation-associated gene 7 (Mda-7) encodes IL-24, which can induce apoptosis in cancer cells. A novel gene therapy approach to treat deadly brain tumors, recombinant mda-7 adenovirus (Ad/mda-7) efficiently kills glioma cells. In this study, we investigated the factors affecting cell survival and apoptosis and autophagy mechanisms that destroy glioma cells by Ad/IL-24.

**Methods:**

Human glioblastoma U87 cell line was exposed to a multiplicity of infections of Ad/IL-24. Antitumor activities of Ad/IL-24 were assessed by cell proliferation (MTT) and lactate dehydrogenase (LDH) release analysis. Using flow cytometry, cell cycle arrest and apoptosis were investigated. Using the ELISA method, the tumor necrosis factor (TNF-α) level was determined as an apoptosis-promoting factor and Survivin level as an anti-apoptotic factor. The expression levels of TNF-related apoptosis inducing ligand(TRAIL) and P38 MAPK genes were assessed by the Reverse transcription-quantitative polymerase chain reaction(RT‑qPCR) method. The expression levels of caspase-3 and protein light chain 3-II (LC3-II) proteins were analyzed by flow cytometry as intervening factors in the processes of apoptosis and autophagy in the cell death signaling pathway, respectively.

**Results:**

The present findings demonstrated that transduction of IL-24 inhibited cell proliferation and induced cell cycle arrest and cell apoptosis in glioblastoma. Compared with cells of the control groups, Ad/IL24-infected U87 cells exhibited significantly increased elevated caspase-3, and TNF-α levels, while the survivin expression was decreased. TRAIL was shown to be upregulated in tumor cells after Ad/IL-24 infection and studies of the apoptotic cascade regulators indicate that Ad/IL-24 could further enhance the activation of apoptosis through the TNF family of death receptors. In the current study, we demonstrate that P38 MAPK is significantly activated by IL-24 expression. In addition, the overexpression of mda-7/IL-24 in GBM cells induced autophagy, which was triggered by the upregulation of LC3-II.

**Conclusions:**

Our study demonstrates the antitumor effect of IL-24 on glioblastoma and may be a promising therapeutic approach for GBM cancer gene therapy.

## Background

The most current and mortal primary adult brain cancer is grade IV astrocytoma or glioblastoma multiforme (GBM). Glioblastoma is a highly aggressive brain cancer with a poor prognosis and low survival rate that is characterized by strong vascular proliferation, invasiveness, diminished apoptosis, and radio and chemo-resistance. Despite treatment, the median survival of GBM patients is approximately 12 months from diagnosis [1]. One of the most difficult challenges in clinical oncology is still treating GBM Understanding the molecular mechanisms of these genetic dysregulations is crucial for the development of more effective diagnostic methods and targeted therapeutic strategies for patients with glioblastoma [2]. Gene therapy is an advanced technology that offers excellent therapeutic potential for different tumor therapy [3]. Tumor destruction is not only accomplished by cell lysis but can also be enhanced by engineering therapeutic genes that directly modulate the tumor microenvironment or tumor cells. The use of different means of transportation, including liposomes and genes, especially cytokines and suicide genes, are among the new treatments. By replicating a recombinant adenovirus containing genes with antitumor function, the replication of genes stored by the virus also increases, leading to higher expression levels in tumor tissues. Previous studies have shown that cytokines such as interleukins (ILs) play an important role in GBM progression [4]. Interleukin-24 (IL-24)/Melanoma differentiation-associated gene-7(Mda-7) is a special member of the IL-10 family of cytokines that can specifically cause apoptosis in cancer cells without affecting healthy cells. The multi-functional tumor suppressing properties of Mda-7/IL-24 include: inducing apoptosis, inhibition of migration, invasion, and eventually metastasis; induction of differentiation and/or killing of cancer stem cells; sensitization of cancer cells to radiation, chemotherapy, and induction of an anti-tumor immune modulating effect [5, 6]. Also, based on the positive results from pre-clinical and Phase I clinical trials, IL-24 has been transitioned into a phase II clinical trial, indicating that it has the potential to be safe and effective for cancer gene therapy. Albeit signaling pathways triggered by IL-24 have been the focus of intensive studies, the mechanisms governing cancer-specific apoptosis triggered by IL-24 are nevertheless not well understood [7]. In the present study, we explored the antitumor properties of a recombinant adenovirus expressing IL-24 as a therapeutic gene in human GBM cell line U87 and its effects on the cell death pathway. These findings are important in our knowledge of IL-24 as a tumor suppressor protein, as well as an immunomodulatory cytokine.

## Materials and methods

### Cell lines

Human GBM cell lines (U87) and AD-293 cell line. U87 is a human glioblastoma cell line commonly used in brain cancer research. (AD-293) that is useful for both packaging and amplification of recombinant adenovirus derived from Human embryonic kidney cells (HEK-293). This cell line was purchased from the National Cell Bank of Iran (Pasteur Institute of Iran, Tehran, Iran). It was cultured in high-glucose Dulbecco’s minimal essential medium with 10% fetal bovine serum), 15 mM HEPES, penicillin (100 U/ml), and streptomycin (100 µg/ml) at 37 °C with 5% CO2.

### Construction of recombinant adenovirus Ad/IL-24

The replication of defective adenovector expressing GFP(Ad/GFP) and adenovirus expressing interleukin-24 or Mda-7(Ad/Mda-7) has been provided as described [8–10] based on AdEasy™ Adenoviral Vector System (Agilent Technologies). Briefly, Mda-7expressing cassette was cloned into adenovector backbone plasmid through homologous recombination at electrocompetent BJ5183 bacterial host. Then the recombinant plasmid was transfected into the HEK-293AD packaging cell and the resultant viral vector particles were harvested, propagated, and finally purified by centrifugation and filtration. The viral vector count was measured by 50% cell line infective dose assay (TCID50), and the Reed-Muench method. The suitable viral MOI (multiplicity of infection) for experimental steps was determined by the number of viruses/cells in which the lowest toxicity was observed 24-hour post-transduction. We used two recombinant adenoviruses to infect U87 cells, one of which contained the interleukin 24 genes (Ad/IL-24), and the other lacked the therapeutic gene and expressed only the GFP gene(Ad/GFP), although GFP expression was common in both vectors. At first, U87 cells were seeded (3.0 × 10^4^ per/well) 1 day before infection. Then, viral vectors used were infected in a 1mL culture medium. Stock virus preparations were diluted in DMEM containing 2% FBS and inoculated onto cell monolayers at the indicated multiplicity of infection (MOI). After 2 h virus adsorption at 37^º^C with rotary agitation for 15 s, every 15 min, DMEM containing 2% FBS was added to the infected monolayer cultures and then incubated at 37 °C for 48 h. GFP positive cells were counted using flow cytometry and a microscope.

### MTT assay

The U87 cell lines were seeded in 96-well plates in each well of plates at a 3.0 × 10^4^ density and were grown in these cell culture plates for 24 h then cells infected with different MOI (3,5, and 10) of Ad/IL-24 for 48 h. Cells without virus infection were used as controls. After incubation with a cell proliferation assay kit (MTT) (Sigma, USA) for 48 h, the medium was removed and the reaction was stopped with dimethyl sulfoxide (DMSO). Finally, the absorbance was measured at 540 nm using an absorbance microplate reader aiming to assess the MTT reduction (Anthos Labtec Instruments, Austria). Each test was checked three times (*n* = 3).

### LDH release measurement

Cell lysis was determined by evaluating the secretion of lactate dehydrogenase (LDH) into a culture medium by LDH assay kit (Takara Bio, Tokyo, Japan), following the protocol of the kit. The U87 cells were seeded at a density of 3.0 10^4^ cells/100 l in 96-well plates and infected for 48 h with Ad/IL-24 at MOIs of 3, 5, and 10. Then, the medium (DMEM) was refreshed and supplemented with 2% FBS, 1% pen/strep, and centrifuged at 250 g/min for 10 min. Then, 100 µl of the supernatant from each well was added to the wells of a fresh 96-well plate. This was done after adding 100 µl of the LDH test solution to each well and incubating the mixture at room temperature for 30 min. Finally, absorbance measurements were taken at wavelengths of 490 and 630 nm, and calculations were made using the kit’s formula. There were at least three runs of this test (*n* = 3).

### Cell apoptosis detection

Ad/IL-24-induced apoptosis of U87 tumor cells was demonstrated by flow cytometry-based Annexin V/7 staining. The Ad/IL-24 effect on the apoptosis of U87 cells was tested via PE Annexin-V/PI Apoptosis Detection Kit (BioLegendend, USA). In summary, 3 × 10^4^ cells were seeded in each well of a 24-well plate. Then, U87 cells were infected with Ad/IL-24 at MOIs of 3, 5, 10 for 1 h, and media was renewed by DMEM containing 1% pen/strep with 2% fetal bovine serum for 48 h. In the first step, after separating the cells from the flask, by adding 2ml of PBS solution at 1500 rpm, we centrifuge for 5 min and wash to remove the cell medium. After washing, the cell sediment is brought to a volume of 500 µl using Binding Buffer 1X. Then add 3 µl anti-annexin V/PI staining antibody with conjugated polyethylene (BioLegend, USA) and incubate for 15 min at 4 °C temperature and in the dark. (We use 7AAD color to correct and adjust the PE and PI color overlap.) After the incubation time, add 1ml of Buffer Binding 1X solution to the tubes and spin at 1500 rpm for 5 min. Centrifuge and add another 500 µl of Buffer Binding 1X to the cell sediment. The wavelength of PE-V Annexin is equal to 578 nm in the H-FL2 channel and the wavelength of the PI dye is equal to 647 nm in the H-FL3 channel in the BD FACS Calibur machine (BD biosciences, San Jose, CA, USA). For flow cytometry, Annexin V staining, the mean ratio of cells stained with Annexin V was compared with the corresponding control group.

### Cell cycle analysis

The U87 cell lines were seeded in 24 well plates in each well of plates at a 3.0 × 10^4^ density and were grown in these cell culture plates for 24 h, then cells infected with different MOI (3,5, and 10) of Ad/IL-24 for 48 h and with the use of the Flow Jo Software (Tristar, CA, USA), the percentages of G0/G1, S, and G2/M stage cells were calculated. Treated and untreated cells were harvested and washed twice with 1× PBS (4,000 rpm, 10 min, 25 °C). Thereafter, cells were fixed with 70% ice-cold ethanol and stored at 4 °C overnight. Then the cells were stained with 1 ml of MASTER MIX PI solution containing 40 µl PI, 10 µl RNase, 950 µl PBS) for 30 min at room temperature in the dark. With the use of the Flow Jo Software (Tristar, CA, USA), the percentages of G0/G1, S, and G2/M stage cells were calculated.

### RNA extraction, cDNA synthesis, and quantitative real-time PCR (qRT-PCR)

For invitro gene expression analysis at first 5 × 10^5^ cells were seeded in each well of a 6-well plate. Then, U87 cells were infected with Ad/IL-24 at MOIs of 3, 5, 10 for 1 h, and media was renewed by DMEM containing 1% pen/strep with 2% fetal bovine serum for 48 h. According to the manufacturer’s instructions, total RNA was extracted from cultivated U87 cells using the High Pure Isolation Kit (Roche, Germany). Complementary DNA (cDNA) was synthesized with total RNA using a reverse transcriptase cDNA synthesis kit (Takara, China). For real-time quantitative PCR (qRT-PCR) analysis, cDNA was amplified using an Eva Green PCR kit (Solis Bio Dyne, Estonia) and the real-time PCR system (Agilent, USA). The differential expression was calculated using the 2^−ΔΔCT^ method and statistically evaluated. Specific primers targeting IL-24/Mda-7, TRAIL (TNF-related apoptosis-inducing ligand), P38 MAPK (Mitogen-activated protein kinase). The β-actin housekeeping gene was also used as an internal control of qPCR reactions. The qPCR conditions were set for 12 min at 95˚C followed by 40 cycles of 15 s at 95˚C, 20 s at 56˚C, and, a final extension of 20 s at 72˚C. The amplification signals of different samples were normalized to the ACTB cycle threshold (Ct), and then the 2^−∆∆ CT^ method was applied for comparing mRNA levels of activated vs. the control (normal U87), which represented a foldchange in data analysis (Table 1).

**Table 1:** Primers used in the present study

**Gene name**	**Primer Sequence (5'‑3')**	**Size (bp)**
IL-24	Forward: AGCTCAGGATAACATCACGAGTG	261bp
	Reverse: CTGTGTGCACTGTCTCTGATGG	
P38	Forward: TTCTACCGGCAGGAGCTGAAC	101bp
	Reverse: GCAGCACACACAGAGCCATAGG	
TRAIL	Forward: AAGTGGCAACTCCGTCAGCTC	164bp
	Reverse: GTGTTGCTTCTTCCTCTGGTCC	
β-actin	Forward: AGACCTGTACGCCAACACAGTG	215bp
	Reverse: CATACTCCTGCTTGCTGATCCAC	

### Cleaved caspase-3 assay

The apoptosis induced by different MOIs of Ad/IL-24 was measured with flow cytometry assay, according to the manufacturer’s protocol. For this purpose, we targeted cleaved caspase-3, which is a credible marker for cells that are dying or have died by apoptosis. Briefly, the cells were labeled with the primary antibody against Cleaved caspase-3 (Cell Signaling, USA) diluted in PBS containing 1% bovine serum albumin (BSA) for 30 min next, cells were washed with PBS, and then PE-conjugated secondary antibody (Anti-donkey IgG, BioLegend, USA) was added and kept at room temperature for 30 min. Finally, the stained cells were examined with flow cytometry (Becton & Dickinson Biosciences, USA). Each experiment was assayed three times (*n* = 3).

### Measurement of TNF-α and survivin concentration by ELISA

The level of tumor necrosis factor (TNF-α**)** as a factor promoting apoptosis and survivin as an anti-apoptosis factor was also determined. The human TNF-α and survivin ELISA kits (Abcam, USA) were used to measure the levels of TNF-α and survivin in viral lysate. For the assay, cells were infected with different MOIs of Ad/IL-24 (MOI: 3, 5, and 10) and cell lysate collected at 48 h. Tumor lysates were filtered via 40 μm filters, centrifuged at 5000 rpm for 10 min at 4 °C. Protein concentration of viral tumor lysate samples were adjusted to 2 mg/ml with PBS and then the level of human TNF-α and survivin was determined using human ELISA Kits (Abcam, USA) according to the producer protocol. Each experiment was assayed three times (*n* = 3).

### Detection of autophagy induction

Induction of autophagy was highlighted by evaluating autophagosomal marker microtubule associated protein 1 light chain 3-II (LC3-II) via specific antibodies using the flow cytometry technique. In the following, cells were suspended and fixed in 4% formaldehyde for 15 min. Then the cells were incubated with 0.2% Triton X-100 for 10 min at room temperature. Then, were labeled with the primary antibody against LC3-II (Abcam, USA) diluted in PBS containing 1% BSA for 30 min. Next, cells were washed with PBS, PE-conjugated secondary antibody (Anti-donkey IgG, BioLegend, USA) was added and incubated at room temperature for 30 min. The stained cells were analyzed using flow cytometry (Becton & Dickinson Biosciences, USA). Each experiment was tested three times (*n* = 3).

### Statistical analysis

Values are expressed as the means ± standard deviation (SD). The statistical analysis of the results was performed using a one-way analysis of the variance (ANOVA) or Student’s t-test. *P* values < 0.05 were considered.

## Results

### Adenoviral vectors selective replication in vitro

**Fig. 1:**
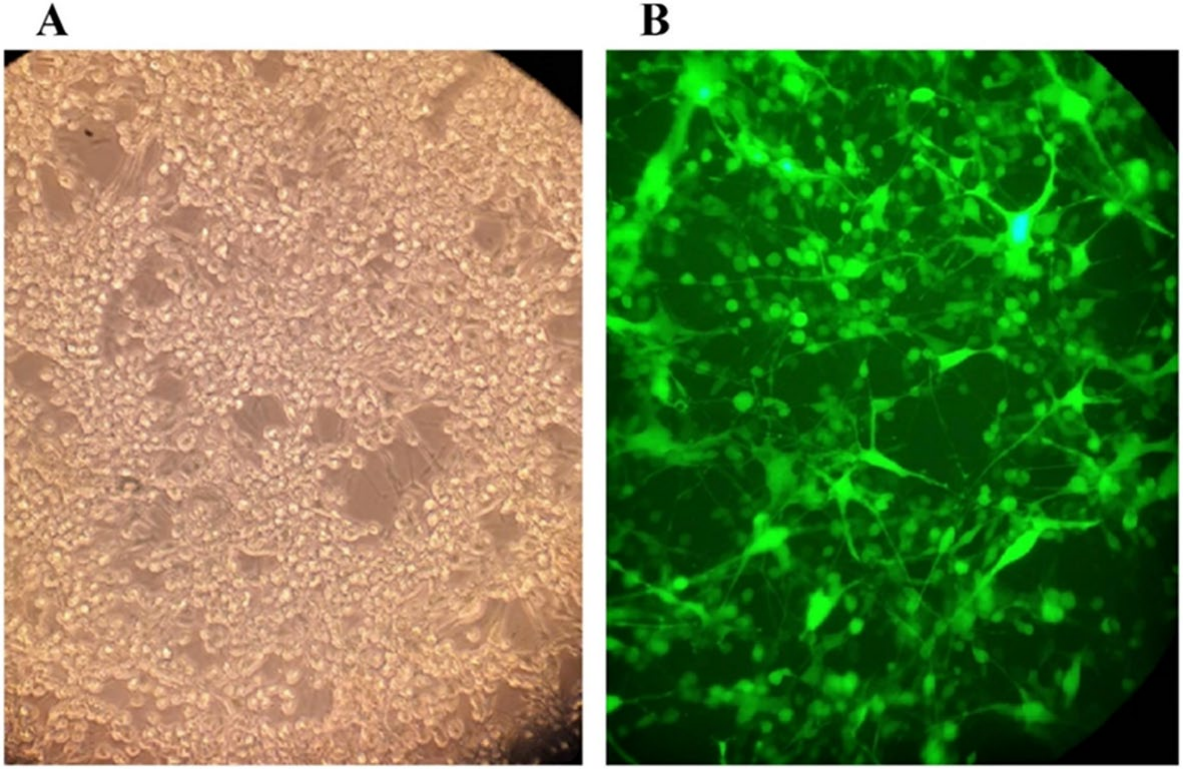
The expression of interleukin IL-24 in U87 transfected with Ad/GFP/IL-24. **A** U87cells were transfected with Ad/GFP/IL-24 and checked for the microscopy visible light. **B** Green fluorescent protein (GFP) expression by fluorescence microscopy

Cells were incubated with the respective adenoviral vectors at MOI 3, 5, and 10. Ad/IL-24 and Ad/GFP had a similar replication capacity in U87 cells. These results revealed that IL-24 and GFP have no effect on selective replication. In U87 cells, Ad/IL-24 and Ad/GFP groups were compared with a fluorescence microscope. As shown in Fig. 1, more than 90% of Ad/IL-24 expression was found in U87 cells. The results of the study of the therapeutic effects of Ad / IL-24 were compared with the control groups including vector control and non-infected cell control. Therefore, we considered a single chart as a vector control along with a cell control for analysis.

### The cytotoxicity effect of Ad/IL-24 using MTT assay

**Fig. 2:**
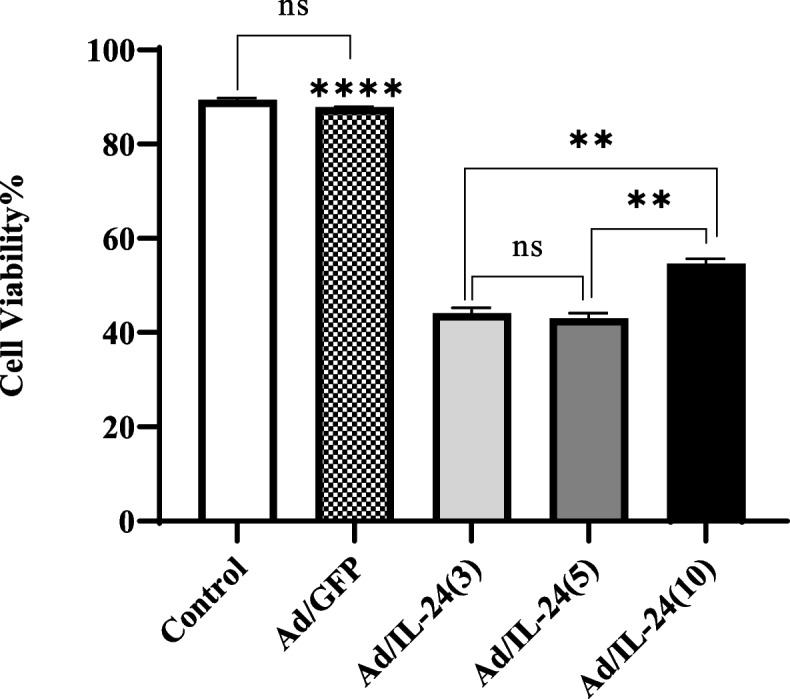
Ad/IL-24 cytotoxicity in U87 cells assessed. The MTT assay results indicated that infection with different MOI can significantly reduce the viability of U87 cells vs control groups (Control and Ad/GFP) ****(*P* < 0.0001), **(*P* < 0.001), indicates a statistically significant difference between MOIs 3,5 and 10 compared with the control group by one-way ANOVA

To determine whether Ad/IL-24 has cytotoxic effects on U87 cells, an MTT reduction assay for various MOI of Ad/IL-24 was applied. Because the MTT reduction of mitochondrial enzyme can only occur in metabolically active cells, the level of activity is measured as cell viability. As shown in Fig. 2, Ad/IL-24 effectively reduced U87 cell viability at MOIs 3,5, and 10 compared with control groups. Cells infected with Ad/GFP and uninfected cells were considered as control groups. Our findings also showed that the survival of U87 cells at MOI 3, 5, and 10 decreased to 44%, 43%, and 54%, respectively.

### The cytotoxic effects of Ad/IL-24 using LDH assay

**Fig. 3:**
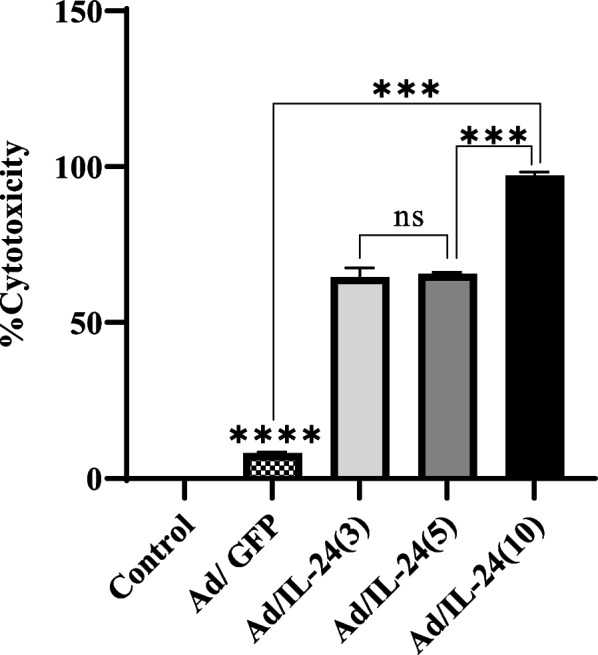
Cytotoxicity of Ad/IL-24 on U87 cells evaluated by LDH assay kit. The LDH assay results indicated that infection with various MOIs can significantly release LDH (all results normalized by uninfected internal control and Ad/GFP). *** *P* < 0.0001, **** *P* < 0.0001. All experiments are done in triplicate and repeated three times

The cell culture medium was quickly damaged during apoptotic or necrotic events due to the rapid cell damage. The cytotoxic effect of Ad/IL-24 was assessed through LDH release after 48 h incubation with different MOI on U87 cells. The LDH activity in the culture supernatant is increased by the growth of dead cells. As demonstrated in Fig. 3 the rate of LDH release in Ad/IL-24 induced U87 cells increased Commercially available kit (Takara Bio, Tokyo, Japan) was used to perform an LDH assay from a culture medium. LDH is an enzyme that is released in an MOI dependent manner than control cells. The results also showed that the toxicity was at concentrations respectively of 3,5 and10 (64.68% ± 2.8), (65.66% ± 0.57) that maximum release, 97.2% ± 1.08) was observed 48 h after infection at an MOI of 10 in the U87 cells (*P* < 0.0001) in U87 cells compared to that with Ad/GFP as control group (15.65% ±2.53).

### The effect of the Ad/IL-24 on apoptosis induction using annexin V/PI

**Fig. 4:**
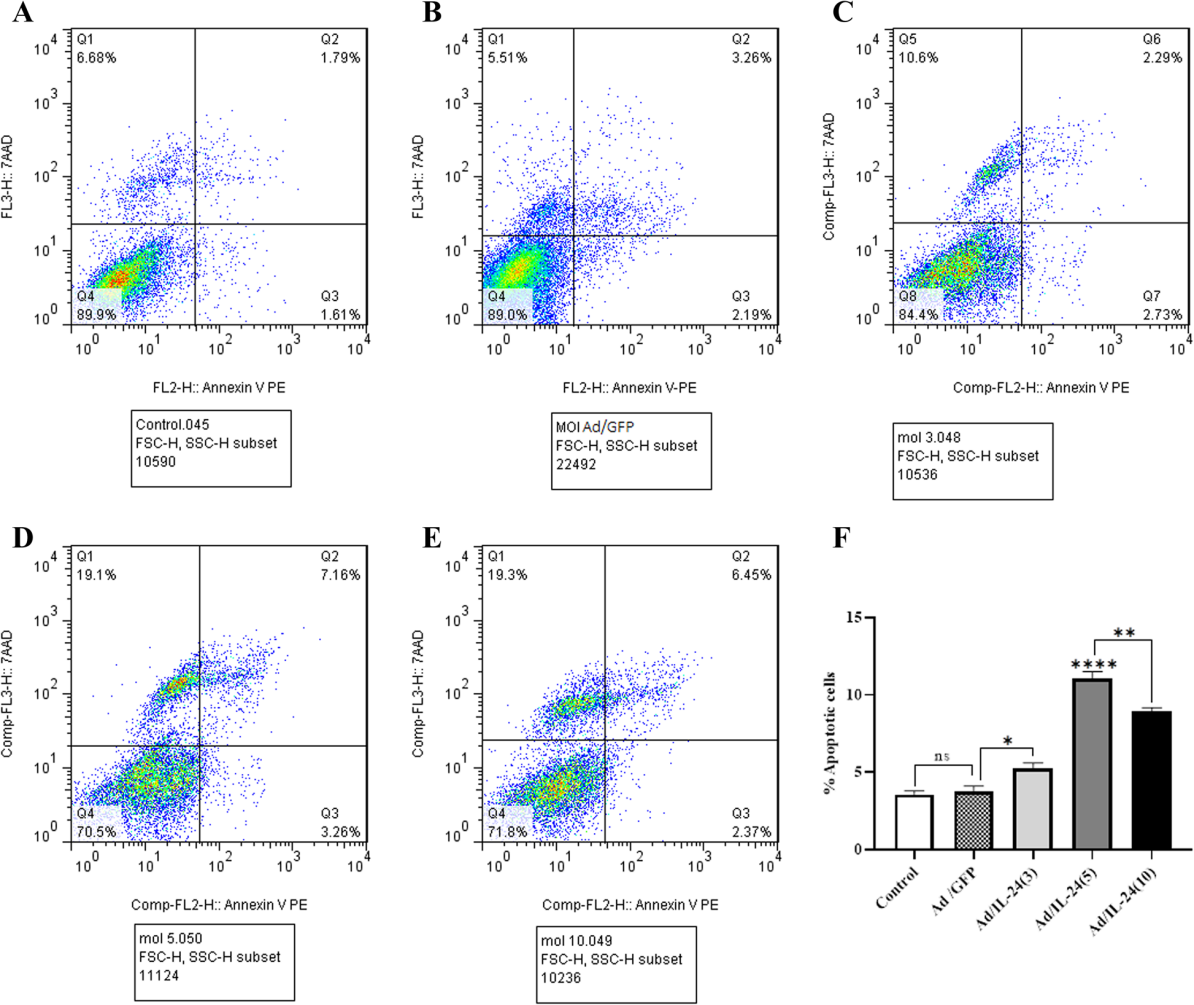
Annexin V/PI staining of U87 cells treated with Ad/IL-24. **A**-**E** U87 cells were treated with various MOI of Ad/IL-24 (3,5 and 10) for 48h, after that expose to annexin V/PI staining and analyzed via flow cytometry. Untreated and Ad/GFP cells were considered as control groups. **F** The total percentage of apoptotic cells was stained with annexin V/PI. *(*P*< 0.0316, **(*P*< 0.008), ****(*P* < 0.0001)

Assessment of the percentage of apoptosis/necrosis induction in Ad/IL-24 treated U87 was conducted by using flow cytometry, via annexin V/PI staining. The means of the percentage ratio of Annexin V positive and PI negative (apoptosis/viable cells) U87 cells were plotted with Ad/IL-24 at different MOIs. As presented in Fig. 4, the percentages of early apoptotic cells were increased to (5.2% ± 0.34), (11.05% ± 0.89), and (8.97% ± 0.21) after 48 h of treatment with Ad/IL-24 MOIs 3,5 and 10 respectively. The results also showed that maximum apoptosis induction, 11.05% ± 0.34 was observed 48 h after infection at an MOI of 5 in the U87 cells (*P* < 0.0001). The results of the experiment indicated that U87cells inoculated with an MOI of 5 provided the highest antitumor effect titer at 48 h post-infection. Results indicates a statistically significant difference between all MOIs compared with the control groups (uninfected internal control and Ad/GFP) by one-way ANOVA.

### Cell cycle analysis

**Fig. 5:**
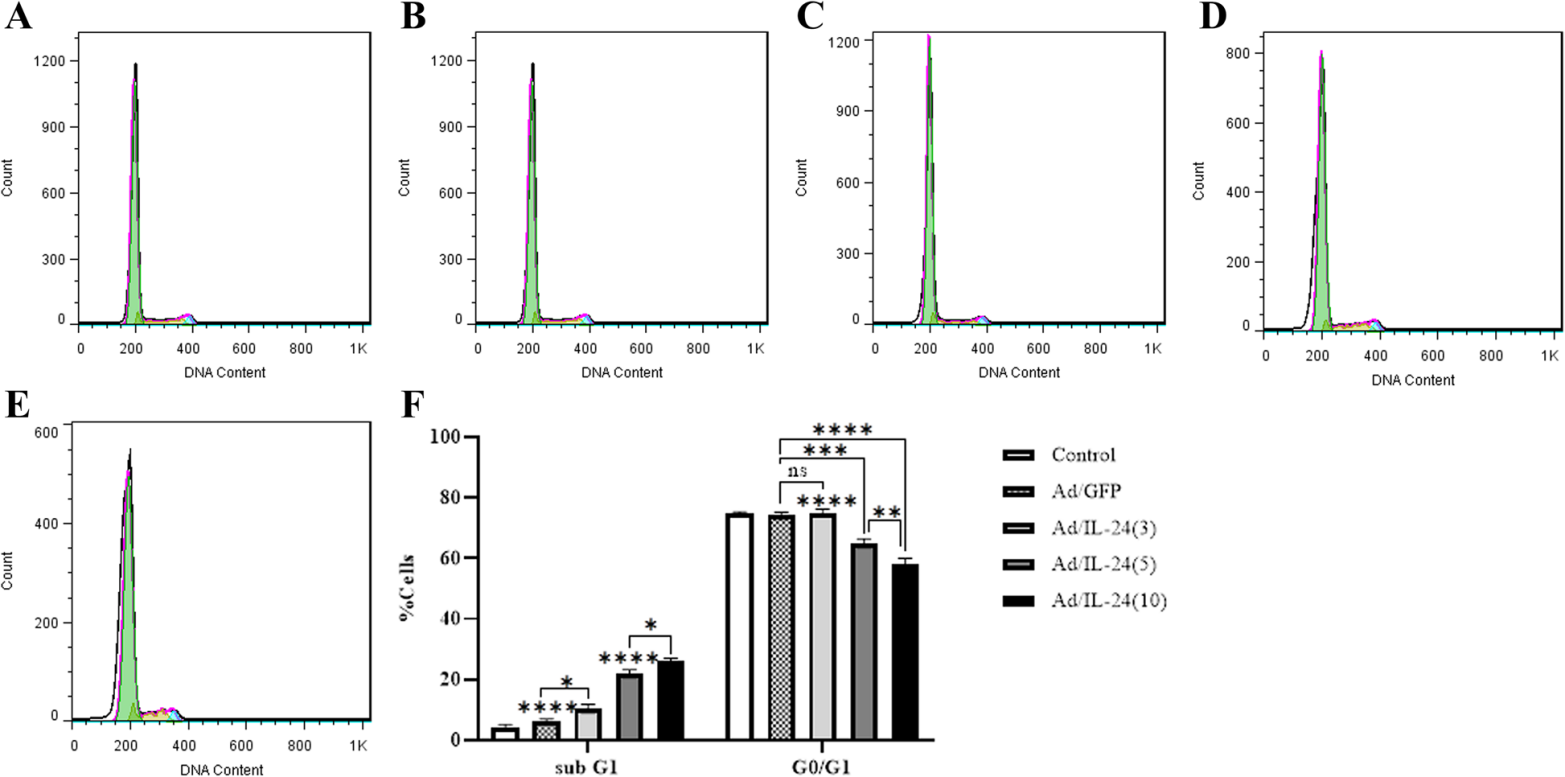
Ad/IL-24 inhibited glioblastoma cells growth in vitro by inducing cell cycle arrest. **A**-**F**: by flow cytometry, U87 cells treated with Ad/IL-24 or Ad/GFP were harvested after 48 h and stained with PI. **F**: Cell cycle distribution was analyzed by flow cytometry and the percentage of cell-cycle phases was analyzed by Flow Jo Software. Each bar represents the mean ±SD of five independent experiments. *(*P*<0.364), **(*P*<0.0027), ***(*P*<0.001), ****(*P* <0.0001)

In order to evaluate the effects of Ad/IL-24 on the cell cycle, U87 MG cells were treated with different MOI for 24 h. DNA content in the sub-G1 phase, G0/G1 phase, S phase, and G2/M phase were determined at the end of the treatment by flow cytometry. Results shows the effects of Ad/IL-24 on the sub-G1 phase and G0/G1 phase in U87 MG cells after 24 h treatment. Untreated cells and cells affected by Ad/GFP showed a normal cell cycle with DNA below G1 Fig. 5F. This increase in the sub-G1 cell population was accompanied by a decrease in the population of cells in the G0/G1phase. Results showed that Ad/IL-24 induced Sub-G1cell cycle arrest in U87 cells compared to that with control cells and Ad/EGFP (Fig. 5) While in the next phase, the number of cells decreased. The number of cells in the Sub G1 phase was (4.11% ±0.97), (6.2% ±0.084), (11.69% ±3.23), (21.45% ± 1.99) and (26.04% ±1.67) in control cell, Ad/GFP and Ad/IL-24 with MOI 3,5,10 groups, respectively. Figure (A-E). On the other hand, the percentage of cells in the G0/G1 phage was (74.85% ±0.417), (74/04% ±1.096), (73.12% ± 3.85), (63.30% ±3.78), and (57.83% ±2.43). Thus, these results revealed that an increase of MOI in Ad/IL-24 led to increased apoptosis in the human GBM cell line.

### Expression of IL-24/mda-7, trail, and P38 MAPK in U87 cells

**Fig. 6:**
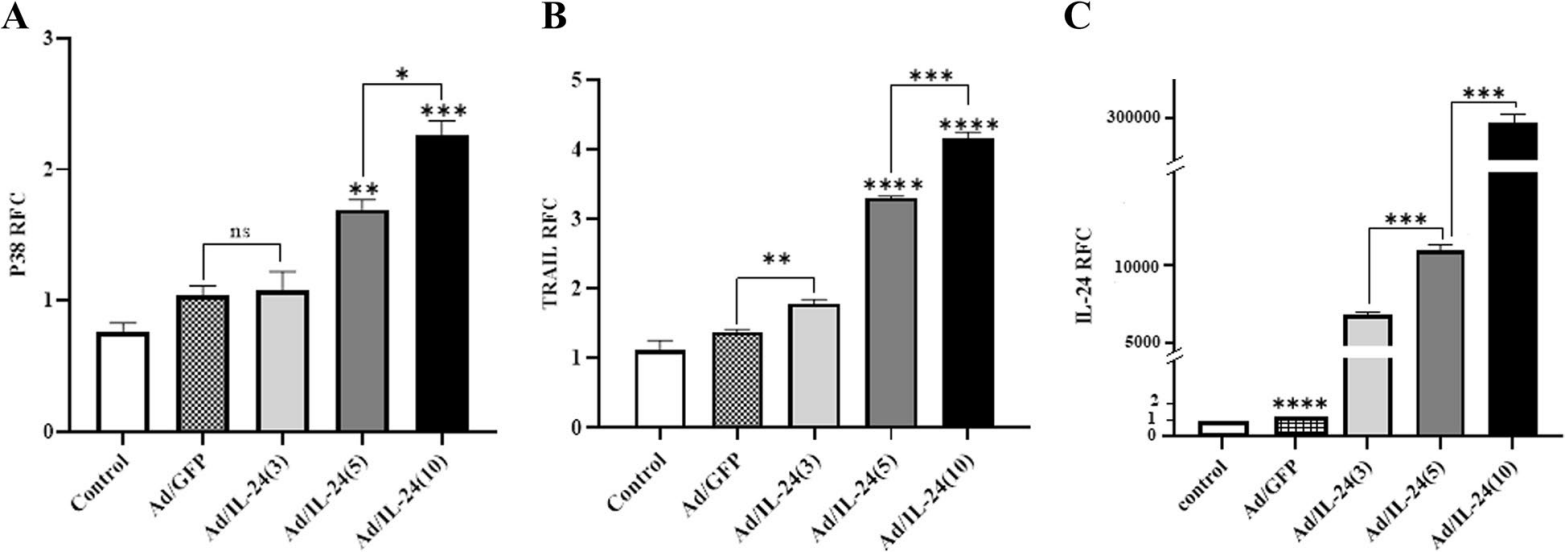
Expression of IL‑24/mda‑7, Trail, and P38 MAPK in U87 cells. **A:** Relative expression levels of P38 MAPK **B:** Trail and **C:** IL-24/mda-7 in U87 as assessed by quantitative reverse transcriptase-PCR. The ratios of expression levels of IL-24/mda-7to β-actin are shown as relative IL-24/mda-7expression levels. Data are presented as mean ± SD.*(*P*<0.02), **(*P*<0.002), ***(*P*<0.0003),****(*P*<0.0001)

IL-24/mda-7, Trail, and P38 MAPK genes as effective factors in the apoptotic process whose expression level was analyzed in U87 cells by the real-time method. Cells were infected at concentrations 3, 5, and 10 with Ad/IL-24. RT-PCR analysis demonstrated that U87 cells express higher expression levels of IL-24/mda-7, TRAIL, and P38 MAPK (2^−ΔΔCT^). Quantitative RT-PCR data showed that P38 MAPK (Fig. 6A) and TRAIL (Fig. 6B) levels were significantly increased in treated cells in an MOI-dependent pattern after 48 h. Analyzes also showed a significant increase of IL-24/mda-7 in treated cells with different MOIs compared with IL-24 Endogenous expression levels in cell line control (Fig. 6C). Our data were considered statistically significant if P-values < 0.05. E) Results indicates a statistically significant difference between all MOIs compared with the control groups by one-way ANOVA.

### Activation of caspase-3 induced by Ad/IL-24 exposure

**Fig. 7:**
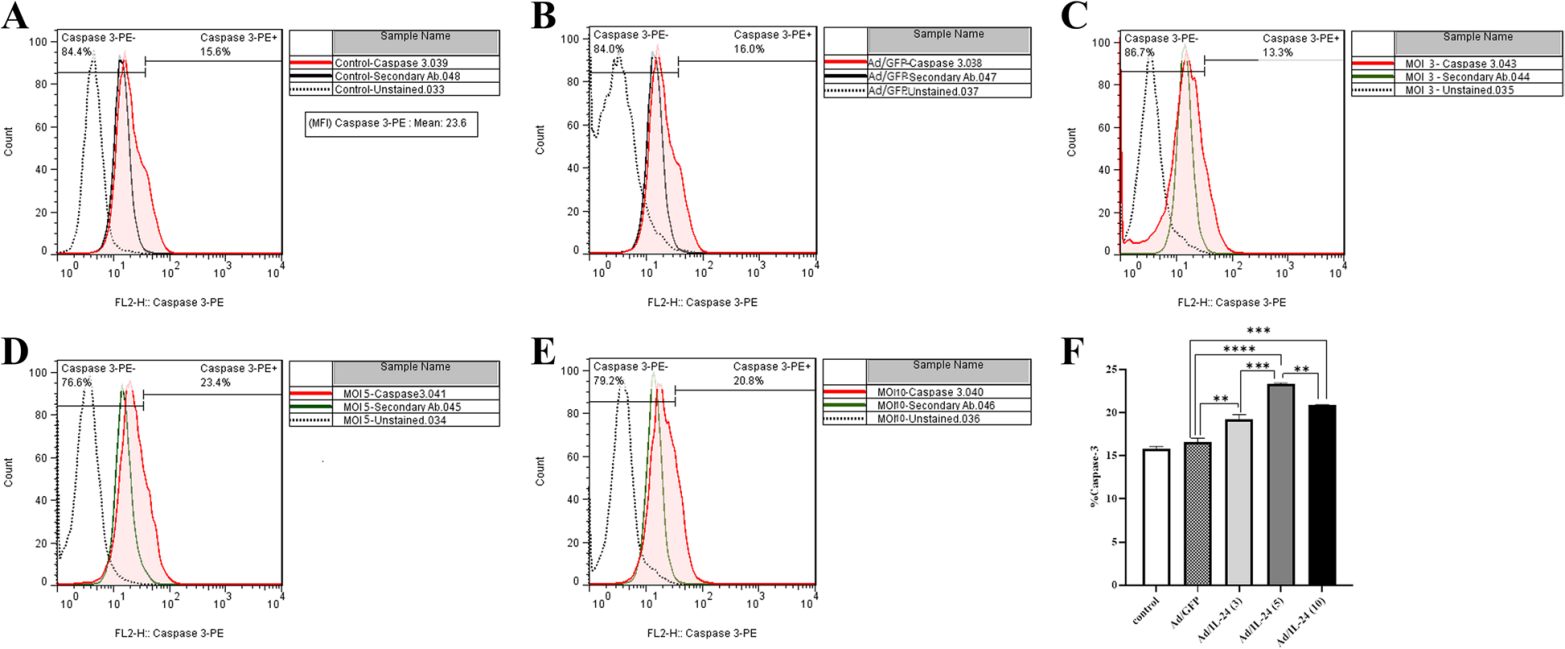
Caspase-3 staining of U87 cells treated with Ad/IL-24. **A**-**E** U87 cells were treated with concentrations of Ad/IL-24 (MOI: 3, 5, and 10) for 48 h, after that expose to secondary antibody staining and analyzed by flow cytometry. Un-treated cells and Ad/GFP were considered control groups. **F** The total percentage of apoptotic cells was stained with Caspase-3. ** (*P* < 0.01), ***(*P* < 0.001), ****(*P*<0.0001)

Caspases are essential mediators during the apoptotic process. In all caspases associated with apoptosis, caspase-3 is a key effector or interpreter of programmed cell death. Therefore, to determine whether caspase-3 was involved in Ad/IL-24-induced cytotoxicity, expression levels of cleaved caspase-3, an indicator of its activity, were examined. In the present study, U87 cells were infected with Ad/IL-24 at MOIs of 3, 5, 10. Then, Ad/IL-24 treatment significantly increased the expression level of cleaved caspase-3 according to the MOI-dependent pattern (Fig. 7). All experiments were done in triplicate and repeated three times (*n* = 3).

### The effect of the Ad/IL-24 on protein levels of TNF-α and survivin

**Fig. 8:**
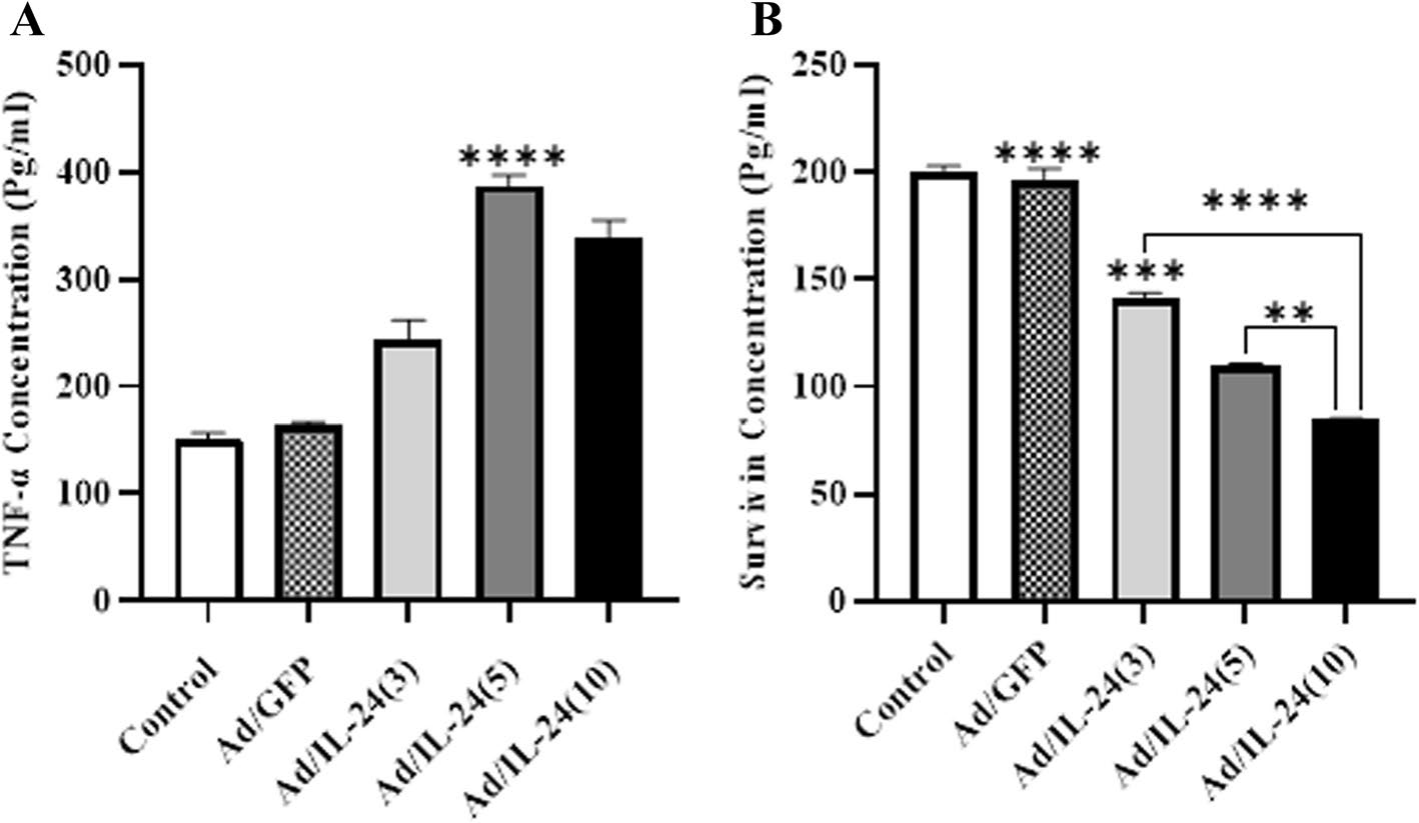
The effect of the Ad/IL-24 on protein levels of TNF-α and survivin. **A** Evaluation of TNF-α and (**B**) Survivin in U87 cell line after transfection with different concentrations of Ad/IL-24 (MOI: 3, 5, and 10) at protein level with ELISA. Un-treated cells consider and Ad/GFP as control groups. TNF-α and Survivin proteins level compared with the control groups **** (*p* < 0.0001), *** (*P*<0.0008), ** (*P*<0023)

TNF-α and survivin protein levels were assessed by ELISA in the Ad/IL-24 treated U87 cell line. As shown in Fig. 8, a significant increase in TNF-α protein level was showed in U87cell line inoculated with MOI 5 as compared to the control groups (*P* < 0.01). In addition, a significant decrease in survivin level was observed in the U87 cell line inoculated with a template-dependent method compared to the control groups. These data suggest that Ad/IL-24 induces the release of DAMPs during lysis of U87 tumor cells, and reduce the level of survivin as anti-apoptosis marker in MOI dependent manner. All experiments were done in triplicate and repeated three times (*n* = 3).

#### Measurement of autophagy activation

**Fig. 9:**
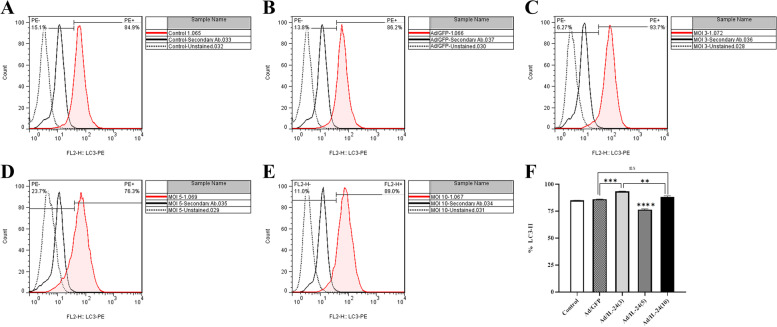
LC3-II staining of U87 cells treated with Ad/IL-24. **A**-**E** U87 cells were treated with different MOIs of Ad/IL-24 (MOI: 3, 5, and 10) for 48 h, after that expose to secondary antibody staining and analyzed by flow cytometry. Un-treated cells consider as control test. **F**. Results of compared with the control groups (Un-treated cells consider and Ad/GFP as control groups. ** (*P*<0.0015), *** (*P* < 0.003), **** (*P*<0001)

The levels of the microtubule-associated protein light chain 3(LC3-II), an auto phagosome protein, were used as an indicator of autophagy. U87 cells were infected with Ad/IL-24 at MOIs 3,5, and 10. then, the expression of LC3-II was examined by flow cytometry 48 h after infection. As shown in Fig. 9, a significant increase in LC3-II protein was detected in the U87 cells infected by Ad/IL-24 at MOIs 3 and 10 compared to the control groups (*P* < 0.05). The un-treated cells and Ad/GFP were considered control groups. These results suggest that infection with the Ad/IL-24 induces autophagy in the U87 cells. All experiments were done in triplicate and repeated three time (*n* = 3).

## Discussion

Gene therapy has gained popularity as a useful method of cancer treatment in recent years. Different cancer treatments might be revolutionized through gene therapy. Numerous disorders have been treated with gene therapy, which has excellent anticancer or cancer suppression benefits [11].

Several approaches have been developed and experimented with in vitro and in vivo for cancer therapy including oncolytic virotherapy, suicide gene-based therapy, cell-mediated gene therapy and inserting tumor suppressor genes, etc. [3, 12]. Safe and efficient delivery of genes is a fundamental and crucial basis for the success of gene therapy, several features have been proposed for effective gene delivery systems. Vehicles must be carefully chosen since they are one of the most important factors in the effectiveness of the therapy [13]. Viral vectors use highly evolved mechanisms that make them suitable both for therapeutic applications and as a tool for biological studies, also they are able to transfer loaded genetic materials into targeted host cells following the natural mechanism of viral infection and multiplication. Many viral vectors have been used for gene delivery, including adeno-associated viruses, lentiviruses, adenoviruses, retroviruses, herpes simplex viruses, etc. Many studies and experimental trials proved that viral vectors confer a higher transduction efficiency than non-viral-based delivery systems with long-term gene expression [14].

Due to the complex nature of cancers, a variety of gene based therapeutic strategies has been used in tumor gene therapy. Studies indicate that loss of Mda-7/IL-24 protein expression correlates with cancer progression and further reinforces the concept that Mda-7/IL-24 functions as a tumor suppressor [15–17]. The cancer selective cell death inducing properties of this therapeutic cytokine, which were demonstrated in a wide range of in vitro studies and in vivo preclinical animal models, make it of significant therapeutic interest. These properties appear to have no detrimental toxicities toward normal cells or tissues [18–20].

Mda-7/Interleukin-24 (IL-24) is a pleiotropic cytokine that has a specific tumor suppression potential that has attracted a lot of attention. It exerts anti-tumor activities mainly through the paths including apoptosis induction, anti-angiogenesis mechanism, cancer stem cell elimination, radio sensitization, and chemo-sensitization. Additionally, Mda-7/IL-24 induces autophagy, bystander effect of the secreted protein, G2/M cell cycle arrest, and inhibition of metastasis [21, 22]. The secreted Mda-7/IL-24 mediates apoptosis in the transduced or neighboring cells through “autocrine” and “paracrine” effects, respectively. Although the detailed mechanism of this differentiation is unclear, one possibility is the difference in homeostasis between a normal cell and transformed cancerous cells [23, 24]. Inherent biochemical differences between normal and cancer cells lead to different responses to IL-24. It was observed that the transformed cells have more ER stress, ROS production, ceramide, low oxygen, or other nutrient levels than normal ones, making them more to apoptosis induction [24, 25]. According to previous research, the Mda-7/IL-24 protein promotes apoptosis in cancer cells in a way comparable to that of endogenously produced protein. In this process, attachment of molecules to the neighboring cells increases the ceramide level, ROS production, and ER stress similar to those for endogenous expression [25].

In the present study, we expanded on these observations and evaluated the effect of overexpression of Ad/ Mda-7 on growth, cell cycle kinetics, and survival of GBM cells. MTT tests and LDH release assays were carried out in order to evaluate and figure out the tumor inhibition titer of Ad/IL-24 with respect to metabolic activity and cellular integrity. The MTT test measures the quantity of purple formazan formed by mitochondrial reduction of tetrazolium salts to identify the metabolic cells. While the release of intracellular contents owing to diminished membrane integrity—apoptotic or necrotic—is indicated by the presence of cytoplasmic lactate dehydrogenase. Our results showed that Ad / IL-24 induced effective antitumor activity against the treated U87 cell line. As shown by annexin V/PI double staining, Ad/IL-24 exerts its antitumor effect through apoptosis, and the highest rate of apoptotic cell production in cell death induction was related to MOI 5.

Studies have shown the induction of G2/M cell cycle arrest by Mda-7 [23, 24, 26, 27]. Another research team showed selective induction of cell cycle arrest and apoptosis in human prostate cancer cells through adenoviral transfer of Mda-7/interleukin-24 (IL-24) [28]. Based on these reports, we investigated the mechanism by which IL-24 induces G2/M cell-cycle arrest in GBM cancer cells. Our results showed that the number of cells in the sub G1 phase of U87 cells treated with Ad/IL-24 increased in an MOI-dependent manner, which indicates an increase in cellular apoptosis. The significant arrest of cells at the sub G1 phase is indicative of apoptosis thus suggesting that Ad/IL-24 act by cell cycle specific mechanism inducing apoptosis in GBM cells.

Previous studies based on gene therapy, has demonstrated that TNF-α gene produces paracrine immune effects against tumors. The researchers showed that high levels of TNF-α are tumor curative, while low levels can be a source of carcinogenesis. They also found that high doses of TNF-α are anti-angiogenic, while low doses induce angiogenesis [29, 30]. TNF-α reduces cell viability and triggers apoptosis, according to certain research [25, 26], either by connecting to its particular receptors on the tumor cell membrane or by activating signaling pathways such Extracellular signal-regulated kinase 1/2 (ERK), c-Jun N-terminal kinase(JNK), p38MAPK, Nuclear Factor Kappa B (NF-kB), and caspase cascades [31]. The findings of the study showed that TNF-α prevents proliferation and induces apoptosis in glioma cells [32]. In the present study, it was shown that U87 cells increased the amount of TNF-α factor after treatment with Ad/IL-24.

Since the functional importance of IL-24 and its relationship with other genes is unknown, we evaluated the effect of Ad/IL-24 on the expression of other genes. Because TRAIL only causes cytotoxicity in cancer cells while sparing normal cells, it is regarded as a potent anticancer agent [33, 34]. In this article, we reported that Ad/IL-24 could obviously induce endogenous expression of TRAIL with an MOI dependent pattern and thus have a positive effect on the apoptosis induction process. Regardless of the mechanism underlying TRAIL induction, we believe that primary tumor killing is due to MDA-7 overexpression and that TRAIL can potentiate the control tumor killing effect. Studies by us and others have demonstrated the activation of multiple signaling pathways by IL-24 in a spectrum of cancer cells resulting in the activation of the caspase cascade and induction of apoptosis [24, 35, 36]. P38 MAPK plays an important role in the regulation of apoptosis, cell cycle arrest, growth inhibition and differentiation. In a similar pattern, p38MAPK activation, up-regulating of caspase-3 level, triggering of Fas/FasL death signal, and mitochondrial dysfunction also start through the bystander effect of traveling IL-24 molecules [25]. The studies organization showed that EGFR signaling complements the self-renewal capability of GSC and their data recommended that the P38 pathway influences survival, cell cycle state, and differentiation popularity of GSC through regulating EGFR trafficking [37]. We found a significant correlation between IL-24 expression level and p38 MAPK gene activation level, which is consistent with previous results. Therefore, in our study, the expression of IL-24 and the resulting increase in the level of p38 in glioblastoma cells can exert its antitumor effects.

Survivin is not always expressed in normal adult human tissues, but is expressed in many human cancers. It has been shown that survivin expression is closely associated with the degree of malignancy and prognosis of patients with cancers [38, 39]. Our research showed that survivin expression levels were significantly decreased in mda-7/IL-24 over expressing cells, indicating that MDA-7/IL-24 is a negative regulator of survivin. Down regulation of Survivin increases the expression of caspases in cells, thus promoting apoptosis [38]. Activation of the family of caspases was known as a crucial mechanism for the induction of death signals to start apoptosis [40]. Caspase-3 is the final step of the apoptosis inducing protease pathway [41]. Studies by the researchers found that caspase-3 expression was significantly increased in tumors transfected with Ad/IL-24 [42, 43]. Our results were consistent with the results of the mentioned articles and the adenovirus containing the IL-24 gene was able to significantly increase the expression of Caspase-3 according to the MOI-dependent pattern. Therefore, there was a significant correlation between survivin and cleaved caspase-3.

Traditional cancer therapy is also mainly targeting enhancing cell apoptosis. However, accumulating evidence suggests that the effects of anticancer therapies are not confined to apoptosis but also involve autophagy. In cancers, autophagy is a “double-edged sword.“ Depending on the types of cells and tissues as well as the stage of the tumor, it can either stimulate or repress tumor development. Generally, the autophagy process can be triggered by both endogenous or exogenous expression of Mda-7/IL-24, but its consequence depends on the cell type by unknown mechanisms. The contribution of autophagy in Mda-7/IL-24 tumor inhibitory roles is controversial. Prior studies in glioblastoma cells showed that GST-Mda-7 treatment caused cell killing via a toxic form of autophagy associated with cathepsin B [44, 45]. GST-Mda-7 caused cell killing by stimulating PERK dependent toxic autophagy in a CD95-independent fashion [46]. Inhibition of autophagy induced by overexpression of Mda-7/interleukin-24 strongly augments the anti-leukemia activity invitro and invivo [47]. To study the mechanism of autophagy, the level of LC3-II protein as an essential autophagy marker that plays separate roles in various stages of autophagosome organization in the U87 cell line after Ad/IL-24 inoculation was evaluated in different MOIs. Our findings showed that Mda-7/IL-24 has a stimulating effect on autophagy, and on the other hand, at 5 MOI, the amount of LC 3-II protein was lower than other MOIs, but it induced apoptosis to a higher extent in U87 cell line. Considering the relationship between these two processes and their mutual influence in the process of cell death, we can be optimistic that by creating a balance between the processes of apoptosis and autophagy, we can take a step in the treatment of brain tumors.

## Conclusion

In summary, adenovirus expressing human IL-24 is able to significantly increase cytotoxicity and inhibit cell growth in the U87 glioblastoma cell line. Overexpression of IL-24 can by increasing factors such as annexin, TNF-α, and increasing TRAIL gene expression induce cell cycle arrest. On the other hand, by increasing the expression of the P38 gene and consequently by increasing caspase-3 protein, LC3-II protein, and decreasing the amount of Survivin, induced apoptosis and cell autophagy. Our study provides a new approach to the treatment of glioblastoma and establishes a theoretical basis for IL-24 as a potential therapeutic gene for GBM cancer gene therapy.

## Data Availability

The datasets used and analyzed during the current study are available from the corresponding author on reasonable request.

## Abbreviations

IL-24: Interleukin-24
Mda-7: Melanoma differentiation-associated gene-7
GBM: Glioblastoma multiforme
HEK: Human embryonic kidney
Ad/IL-24: Adenovirus containing interleukin-24
DMEM: Dulbecco's Modified Eagle Medium
BSA: Bovine serum albumin
LDH: Lactate dehydrogenase
ELISA: Enzyme-Linked Immunosorbent Assay
TNF-α: Tumor necrosis factor- α
MAPK14: Mitogen-activated protein kinase
NF-kB: Nuclear Factor Kappa B
cDNA: Complementary DNA
TRAIL: TNF-related apoptosis-inducing ligand
RT‑qPCR: Reverse transcription‑quantitative polymerase chain reaction
MOI: Multiplicity of infection
LC3-II: Protein light chain 3-II
GSC: Glioma cancer stem-like cells
EGFR: Epidermal growth factor receptor
DMSO: Dimethyl sulfoxide
pen/ strep: Penicillin/ streptomycin
